# Transcriptional Profiling of Formalin Fixed Paraffin Embedded Tissue: Pitfalls and Recommendations for Identifying Biologically Relevant Changes

**DOI:** 10.1371/journal.pone.0035276

**Published:** 2012-04-17

**Authors:** Matilda Rentoft, Philip John Coates, Göran Laurell, Karin Nylander

**Affiliations:** 1 Department of Medical Biosciences/Pathology, Umeå University, Umeå, Sweden; 2 Tayside Tissue Bank, Medical Research Institute, Ninewells Hospital and Medical School, University of Dundee, Dundee, United Kingdom; 3 Department of Clinical Sciences/Otorhinolaryngology, Norrlands University Hospital, Umeå, Sweden; University of Connecticut Health Center, United States of America

## Abstract

Expression profiling techniques have been used to study the biology of many types of cancer but have been limited to some extent by the requirement for collection of fresh tissue. In contrast, formalin fixed paraffin embedded (FFPE) samples are widely available and represent a vast resource of potential material. The techniques used to handle the degraded and modified RNA from these samples are relatively new and all the pitfalls and limitations of this material for whole genome expression profiling are not yet clarified. Here, we analyzed 70 FFPE tongue carcinoma samples and 17 controls using the whole genome DASL array covering nearly 21000 genes. We identified that sample age is related to quality of extracted RNA and that sample quality influences apparent expression levels in a non-random manner related to gene probe sequence, leading to spurious results. However, by removing sub-standard samples and analysing only those 28 cancers and 15 controls that had similar quality we were able to generate a list of 934 genes significantly altered in tongue cancer compared to control samples of tongue. This list contained previously identified changes and was enriched for genes involved in many cancer-related processes such as tissue remodelling, inflammation, differentiation and apoptosis. Four novel genes of potential importance in tongue cancer development and maintenance, SH3BGL2, SLC2A6, SLC16A3 and CXCL10, were independently confirmed, validating our data. Hence, gene expression profiling can be performed usefully on archival material if appropriate quality assurance steps are taken to ensure sample consistency and we present some recommendations for the use of FFPE material based on our findings.

## Introduction

Transcriptional profiling by DNA microarray analysis is proving to be a powerful tool in cancer research, significantly increasing our knowledge of tumour development and progression. It has also provided novel treatment targets and prediction models for prognosis and treatment response [1]–[4]. An obstacle to the widespread use of expression profiling has been the limited availability of fresh frozen (FF) samples from which high quality RNA can be extracted [3]. This becomes particularly important when attempting to discover differences associated with individual sub-types of cancer. For example, although approximately 350 cases of cancer in the oral cavity are diagnosed in Sweden every year, there is an increasing awareness that tumours of different locations within the oral region are not comparable, having different clinical presentations and outcomes and being associated with distinct risk factors and genetic changes [5]–[10]. Thus, the number of fresh samples from individual sub-sites becomes rather limited.

Recent developments have opened up new opportunities to analyze partially degraded RNA from formalin fixed paraffin embedded (FFPE) samples, the standard method for preserving tissue and for which millions of patient samples are stored around the world [11]–[14]. The focused DASL array targeting 502 cancer related genes was the first microarray provided by Illumina designed to handle partially degraded RNA [13]. This array has been successfully used in two studies on oral cavity tumours, one focusing on tongue and the other on buccal mucosa [15], [16]. The results of these analyses provided a high degree of consistency of identified genes [17], which may relate to the focused nature of the interrogated set of genes for general cancer changes. However, identifying site-specific changes in gene expression requires the ability to profile a larger set of genes including those with specific rather than general actions in cancer. The focused array has recently been expanded into a whole genome (WG) array covering 20818 genes [14] and has so far been utilized in a handful of studies on FFPE material, primarily on breast tissue [18]–[20]. One study on oral cancer tissue using the WG-DASL array has been performed comparing tumours with different invasive patterns using formalin fixed and paraffin embedded fresh frozen samples from which RNA was extracted within 24 hours of fixation [21].

Here we analysed FFPE samples from 70 patients with squamous cell carcinoma (SCC) of the tongue that had been stored between 1 and 13 years and evaluated performance of the whole genome DASL array. We then designed the most appropriate set-up for an efficient and accurate differential expression analysis and found 934 genes with altered expression in tumours. To our knowledge we are the first to analyse whole genome gene expression patterns in a large number of FFPE SCC tongue samples that have been archived over a longer period of time, opening up this approach for additional studies into the pathobiology of oral cancer in individual sites and the identification of additional biomarkers.

## Methods

### Sample characteristics and RNA extraction

FFPE blocks from 70 patients with squamous cell carcinoma (SCC) of the moveable tongue between 1997 and 2010 were available for analysis. Patient age varied from 19 to 88 years (mean 58 years). Fifty patients had a T1 or T2 tumour while the remaining 20 patients had a T3 or T4 tumour. Twenty patients had cervical nodal spread at diagnosis. The male to female ratio was 1.3∶1. Seventeen control samples were derived from patients with non-malignant changes on the tongue. The mean age was 49 years and the male to female ratio 1.1∶1 in the control group. Average age and stage distributions in the 28 SCC samples selected for differential gene expression analysis were similar to the whole group but the proportion of women was increased (1∶1.3).

Ten 5 µm sections were collected from each of the 87 FFPE samples and RNA extracted immediately using the high pure RNA extraction kit (Roche Diagnostics GmbH). RNA was eluted in 40 µl elution buffer and a total of 400 ng RNA was needed for pre-qualification and array analysis. Ten paired fresh frozen (FF) samples derived from patients with tongue SCC and adjacent clinically normal oral mucosa were available for qPCR confirmation and RNA was extracted using the trizole method. FF samples came from eight men and two women, all but one had T1 or T2 tumours and average age was 67 years. All RNA samples were stored at −80°C until further use. The study was approved by the local ethical review committee “Etik Provnings Namnden" (EPN), Permit number 08-003 M. For the FF samples written consent was obtained from all patients. For the archival FFPE samples written consent is available for samples from 2003 and onwards because of the establishment of the Swedish Act on Biobanks (SF 2002:297). The use of FFPE samples dated before 2003 was approved by the local ethical review committee (EPN) according to their standard procedure. All samples came from Biobank VL (Vasterbottens Lan).

### Quality measurement and confirmation using qPCR analysis

cDNA reactions were performed using RevertAid H minus first strand cDNA kit (Fermentas) with 200 ng input RNA. For qPCR quality assessment of RNA from FFPE samples the expression of TUBA6, previously shown to be stably expressed in oral tissue [22], was analysed in all FFPE samples as well as two normal FF samples using IQ SYBR green supermix (BioRad) according to the manufacturer's instructions. Quality measurement of the samples (Ct_diff_) was defined as the difference in cycles taken to reach the threshold for the FFPE sample compared to the two FF samples (average) (Ct_diff_ = Ct_FFPE_−Ct_FF_). A difference of more than twelve cycles between the two tissues was set as a cut-off for inadequate RNA quality based on Illumina recommendations. qPCR confirmation of four selected genes was performed using the quanti tect primer assay together with the quanti tect SYBR green assay (Qiagen). Expression levels of the four genes were evaluated in ten of the FFPE samples for which sufficient RNA was available and the ten paired FF samples.

### Array hybridisation and analysis

**Figure 1:**
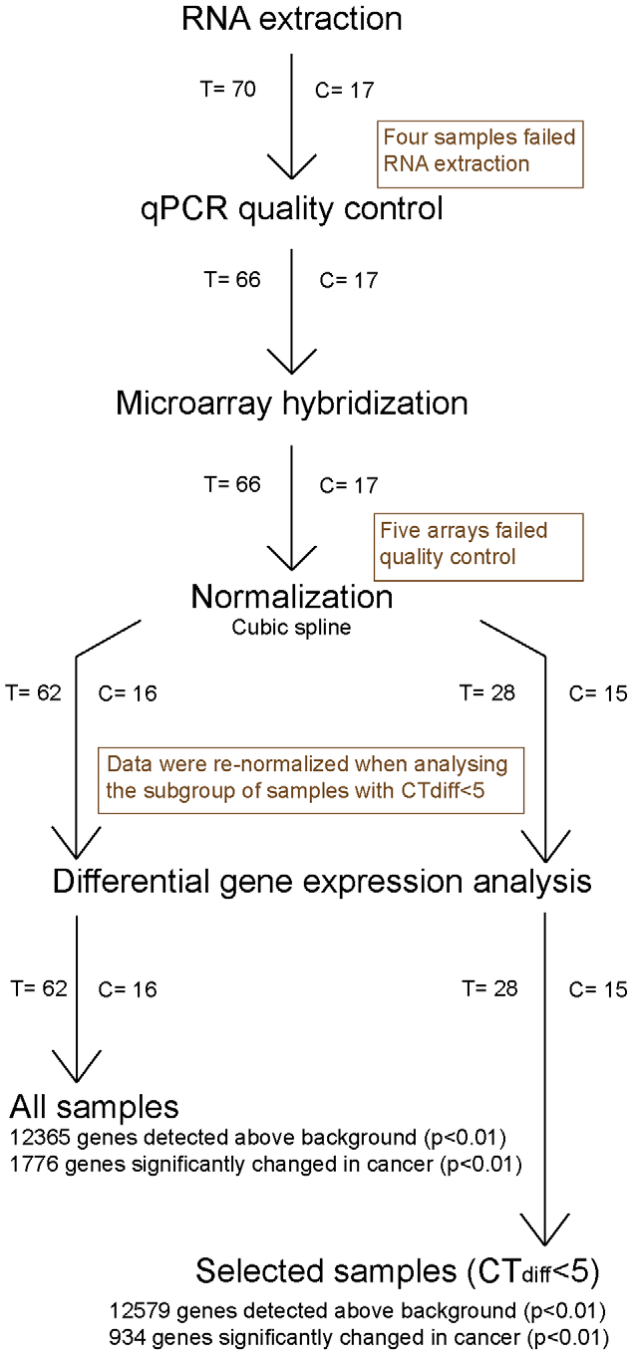
Flowchart illustrating experimental procedure. Description of the procedures used to assess samples from RNA extraction to acquiring of gene lists, including samples removed following each step of analysis. Tumour samples are abbreviated T and controls C.

The DASL assay was performed as previously described [15] with the exception that the WG DASL pool of oligonucleotides was used (containing 29377 probes, covering 20818 genes) and the PCR products precipitated and hybridized to BeadChips instead of the sentrix universal Array Matrix (Illumina). Samples were evenly spread over eight BeadChips which could hold twelve samples each and all samples were run at the same occasion. The BeadArray Reader 500 was used to scan the arrays, and image analysis performed using GenomStudio (Illumina). The average signal and the 95^th^ percentile of the probe intensities (p95) were used to evaluate the quality of the hybridisation. Average signal intensity >500 and a p95 >2500 were judged as acceptable. Several hundred negative control probes were included on the arrays for calculations of detection p-values. Genes significantly detected in either controls or tumours with a p<0.01 were included in the analysis. Raw data were normalized using cubic spline algorithm without background normalization and used in all analyses if not otherwise stated. Array data have been submitted to and are available from the gene expression omnibus (GEO) (accession number GSE34115). The experimental procedure is summarized in a flowchart in Figure 1.

### Statistics

Replicate reproducibility was analysed using simple linear regression and the coefficient of determination (R^2^) and p-values are presented. Linear regression analysis was also used to investigate factors influencing the quality of the sample, the number of genes detected on the array (*i.e.* genes with expression levels significantly above background; p<0.01) and the expression level of individual genes. All regression models only included tumour samples to avoid the large variation caused by the many differentially expressed genes between control and tumour samples. Comparison of quality measurements between tumours and controls and evaluation of qPCR results and sequence differences was performed in SPSS using the appropriate statistical test, as denoted in each figure, and significance was set to p<0.05. Since normality could not be assumed, non-parametric tests were used. Mann-Whitney U-test was used for unpaired samples and Wilcoxon matched pairs signed rank test was used for paired samples. Sequence analysis was performed on probe level while all other analyses were performed at the gene level. When selecting the 10 probes least affected by sample quality, an additional restriction was included, that the gene should be expressed at least 4×background in all samples, to exclude genes that were not recognised as affected because they were close to the detection limit. Differential gene expression analysis was performed using GenomeStudio provided by Illumina. P values were corrected for multiple testing using Benjamini-Hochberg and the significance level set to p<0.01 to increase stringency. Unsupervised clustering of samples was performed using Pearson correlation as the measurement of similarity. Pathway analysis of genes significantly changed in tumours was carried out using GeneGo.

## Results

### Sample and array performance

RNA for microarray analysis was extracted from 70 tongue carcinoma samples and 17 controls. Four tumours provided less than 400 ng total RNA and could not be analyzed further (figure 1). The remaining 66 tumour samples gave on average 3.7 µg of RNA whereas extractions from controls resulted in consistently lower amounts (1.8 µg, p = 0.01) (Table 1).

**Table 1:** Quality measurements of FFPE controls and tumours.

	Group	n	Average	STDEV	p-value^a^
RNA (µg)	C	17	1.8	1.6	0.01
	T	66^b^	3.7	3.4	
Ct diff	C	17	3.5	3.3	0.002
	T	65^c^	5.2	2.2	
Mean signal	C	16^d^	1485	421	0.03
	T	62^d^	1227	359	
p95	C	16	8020	2101	0.06
	T	62	6801	1976	
Detected genes	C	16	11836	1879	0.02
	T	62	10558	2186	

a Mann-Whitney U test.

b 4 samples did not generate sufficient RNA (<40 ong).

c 1 sample failed the PCR reaction.

d 4 tumours and 1 control failed to fulfil array requirements.

**Figure 2:**
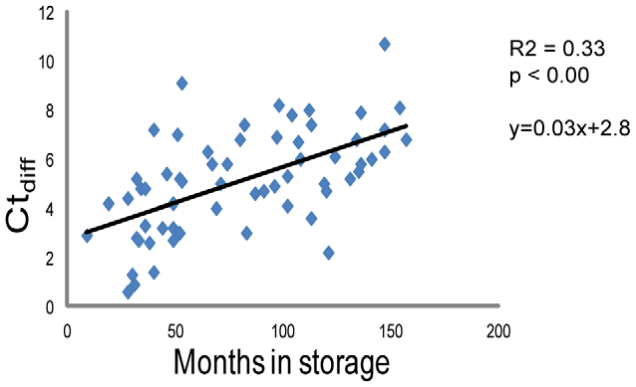
Linear regression analysis. Regression model describing how much of the variation in sample quality (Ct_diff_) can be explained by sample storage time prior to extraction.

The quality of RNA from the FFPE samples was evaluated according to Illumina recommendations by comparing how well they amplified a house-keeping gene using q-PCR compared to RNA from fresh frozen (FF) tissue (Ct_diff_ = Ct_FFPE_−Ct_FF_). One sample failed the PCR reaction while all other FFPE samples reached the threshold between 1–10.7 cycles later than FF tissue, which is within the acceptable level (Table 1). A significant relationship between month in storage and Ct_diff_ was identified using linear regression analysis explaining approximately 33% of the variation (r^2^ = 0.33, p<0.001) (Figure 2).

**Figure 3:**
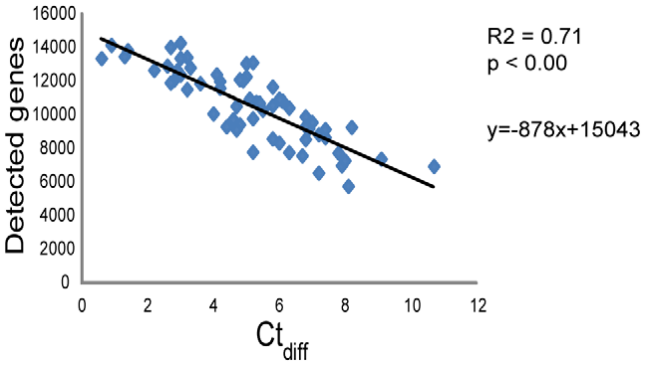
Linear regression analysis. Regression model describing how much of the variation in number of detected genes on the array can be explained by sample quality after extraction (Ct_diff_).

Eighty three unique samples and two replicate samples were hybridized to the arrays, including the sample that failed the PCR reaction. One of the two replicates was placed on the same chip as its counterpart and the other placed on a separate chip to evaluate differences in reproducibility within and between chips. Both replicates showed high correlation (r^2^ = 0.97 and r^2^ = 0.98 respectively). The success rate of the arrays was high and more than 95% passed the quality control. However, five samples, including the sample that failed in the PCR reaction and one control sample, had an average signal intensity <500 and a p95 <2500 and were thus excluded from further analysis, leaving 78 samples (Figure 1). On average, 52% of the genes on the array could be detected. In general, control samples performed better than tumours, with significantly lower Ct_diff_ and higher average signal, p95 and number of detected genes. Data for RNA and array quality measurements are summarised in table 1. The number of genes detected in each sample varied and linear regression showed that this could be explained largely by the quality of the samples measured by Ct_diff_ (r^2^ = 0.71, p<0.001) (Figure 3). Time in storage also explained some of the variation in the number of genes detected (r^2^ = 0.36, P<0.001) but multivariate linear regression indicated that this factor provided very little unique information beyond the contribution of Ct_diff_. Taken together, a strong linear relationship between quality of the RNA and the performance of the arrays was observed. The effect of reduced sample quality therefore leads to a decrease in the average signal intensity of the array and progressively increases the number of genes whose signal intensities fall to background levels, where an increase of one cycle in Ct_diff_ leads to approximately 880 additional genes being classified as not-detected in the poorer quality sample.

### The influence of sample quality on individual genes

**Figure 4:**
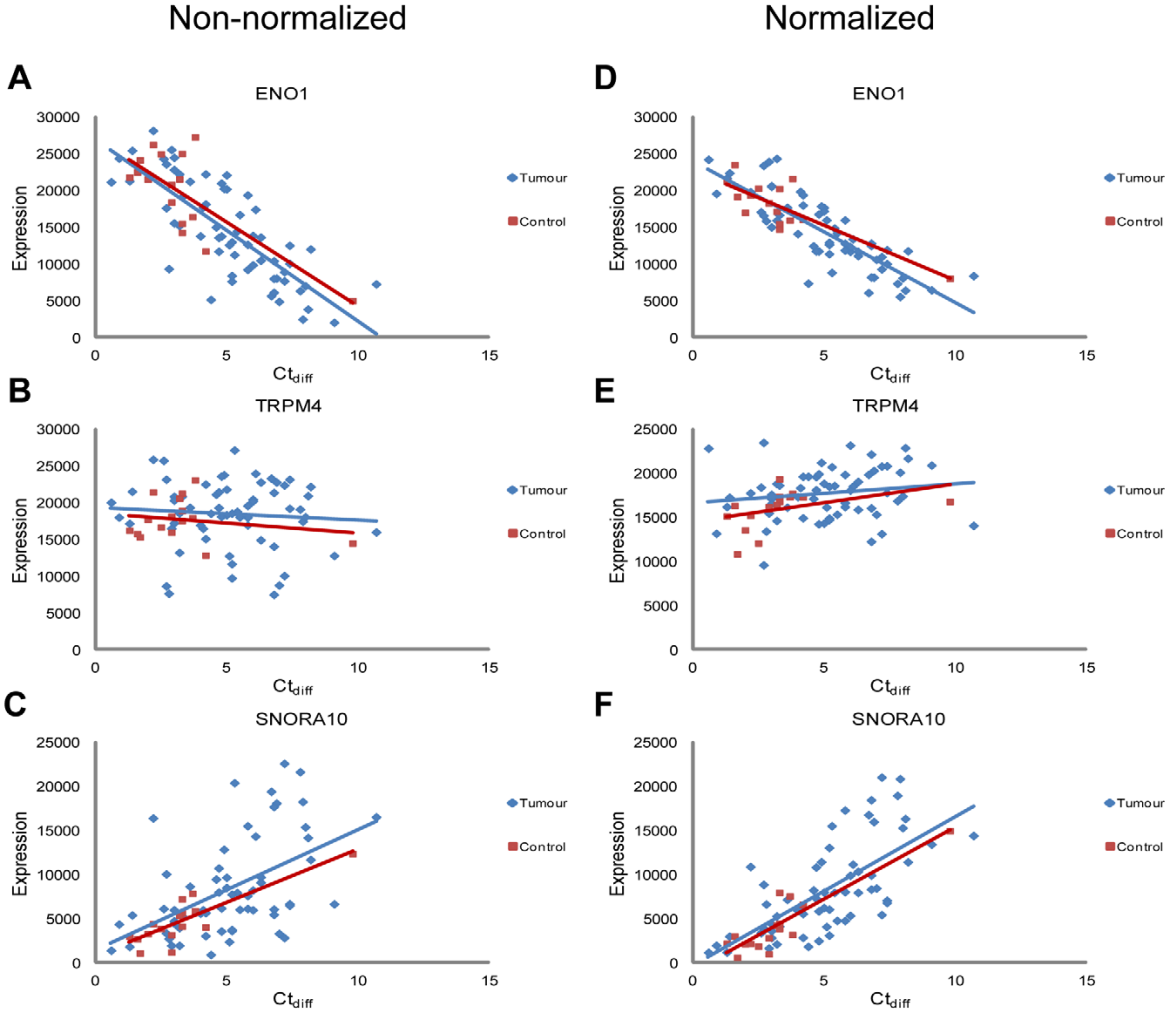
Example of three genes and how their expression was affected by difference in sample quality (Ct_diff_). (A), (B) and (C) Linear regression analysis of the genes YPEL5, TRPM4 and the short non-coding gene SNORA10 describing the relationship between expression of the gene and sample quality (CT_diff_). All 78 samples are included and analysis was performed using non-normalized data. (D), (E) and (F) Linear regression analysis of the same genes using normalized data. Samples and regression line for tumours are denoted in blue and sample and regression line for controls in red.

To investigate how the expression level of individual genes was affected by sample quality a linear regression analysis was performed on each gene detected on the array, asking the question if Ct_diff_ significantly influenced its' expression level. Using non-normalized array data, expression of 60% of the genes was significantly influenced by sample quality. Normalizing data using any of the three methods provided in the Illumina software GenomeStudio at best decreased the number of affected genes to 55%. For non-normalized data, the expression of the majority of the affected genes decreased with a decrease in Ct_diff_, as noted above. In addition, a small percentage (1.8%) of genes had an increased expression with a decrease in quality. Small RNAs, including small nucleolar RNAs (snoRNA) and microRNAs (miRNA), were highly over-represented among those genes. In general, fewer small RNAs were significantly associated to sample quality (32%) and of these 78% showed increased expression with a decrease in sample quality. Figure 4 shows an example of three genes, one whose expression level decreased with a ten cycle change in Ct_diff_ (YPEL5), one that was not influenced by sample quality (TRPM4) and one that had an increased expression with a ten cycle change in Ct_diff_ (SNORA10). The same trend was seen both in control and tumour samples and did not change after normalization.

### Importance of probe sequence for gene expression level

The sequence of the probe could give clues to why there is a difference in how individual genes are affected by sample quality [11], [19]. Comparing the probe sequence of the ten genes that were influenced by sample quality with the highest significance to the ten least affected genes, the total number of Guanines was higher and the total number of Cytosines was lower in the affected genes. In addition, the occurrence of more than two consecutive Guanines was higher in affected genes. Comparing all genes that were significantly affected to the remaining genes gave the same result (Table 2).

**Table 2:** Average number of A,C,G,T and occurrence of two or more consecutive Gs in probe sequence for the 10 most and the 10 least affected genes and p values for all affected compared to remaining genes.

	Influenced genes (n = 10)	Non-influenced genes (n = 10)	p-value (20 genes)^a^	p-value (all genes)^a^
A	10.3	10.7	0.85	0.134
C	11.3	14.1	0.04	9.9E-28
G	15.9	11.9	0.02	1.5E-30
T	12.5	13.3	0.68	0.05
≥GG	4.3	2.0	0.02	2.6E-14

a Mann-Whitney U test.

### Differential expression analysis comparing tumours to controls

**Figure 5:**
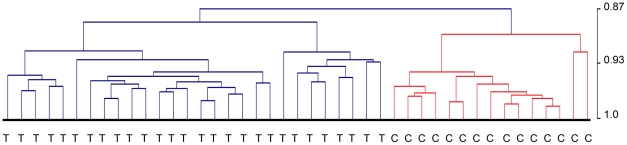
Dendogram from unsupervised hierarchical clustering. Including the 43 samples selected for differential gene expression analysis and all 12579 genes detected in these samples. Control samples are denoted by C and tumours by T. Pearson correlation was used as a measurement of similarity.

As sample quality has an impact on the expression level of some but not all genes, it is important that groups to be compared using differential gene expression analysis include samples of the same quality range. This restriction will ensure that the expression of a gene is not lower or higher only because of a difference in quality between groups. In our data, controls were of significantly better quality. All controls except one had a Ct_diff_ below 5 cycles, which was therefore used as the cut-off value for all samples and 28 tumour samples fell within this range (Figure 1). Unsupervised hierarchical clustering of the 12579 genes significantly detected in these 43 selected samples showed a clear separation between tumour and control samples (Figure 5). Tumours subsequently separated into three distinct clusters but neither gender, age of patient, T-stage or N- stage was significantly associated with any of the groups. Of all detected genes, 934 were found to be significantly changed in tumours and 756 of these were differentially expressed by more than two fold (Table S1). Analysing the 934 changed genes using GeneGO to find over-represented cell functions and pathways showed that the majority of the 12 significant processes detected are important in tumour development and maintenance (e.g. cell cycle regulation, immune response, apoptosis, cell differentiation, vascularization and DNA damage) (Table 3). Performing a similar analysis but including all array samples that passed the standard quality controls (62 tumour and 16 controls; Figure 1) resulted in a poorer separation between tumour and control samples, where one of the controls clustered with the tumours and two of the tumours clustered with controls. The list of differentially expressed genes was also changed into a longer list containing 1776 genes including for example YPEL5 and SNORA10. As can be seen in Figure 4 the difference between tumour and control for these genes is mainly an artefact caused by a difference in quality between tumour and control samples.

**Table 3:** Pathway analysis of significant genes.

Cellular process	p-Value
Immune system response	1.1E-29
Inflammatory response	9.6E-16
Tissue remodeling and wound repair	8.4E-6
Apoptosis	4.2E-5
Cell differentiation	8.1E-3
Cell cycle and its regulation	1.8E-2
Blood clotting	2.8E-2
Cystic fibrosis disease	3.1E-2
DNA-damage response	3.1E-2
Mitogenic signalling	3.2E-2
Transcription regulation	4.4E-2
Vascular development	4.9E-2

### Confirmation of selected genes

Four genes from those identified using only the high quality samples were chosen for confirmation based on their potential importance in tumour development and progression without previously being specifically connected to SCC of the tongue. SLC2A6 and SLC16A3 code for transporters important for cellular energy supply shuttling glucose and monocarboxylic acids in and out of cells and CXCL10 is a chemokine. The precise function of the novel protein SH3BGRL2 is unknown but it belongs to a family of thioredoxine-like proteins [23]. All genes showed good reproducibility when analyzing ten of the FFPE samples using a second method, qPCR. The genes were also analyzed in ten paired fresh frozen samples. SLC2A6, CXCL10 and SLC16A3 were significantly up-regulated and SH3BGRL2 significantly down-regulated, in agreement with the array data. While some genes (e.g. SLC2A6, CXCL10) show a very high fold change (>30 fold) SLC16A3 levels were only on average 1.6 fold higher in FF SCC of the tongue (Table 4).

**Table 4:** Confirmation of four genes in 20 fresh frozen sample using qPCR.

	Array FFPE (28T, 15C)	Fold	p-value	qPCR FF (10T, 10C)	Fold	p-value^a^
SLC2A6	↑	2.4	6.5E-05	↑	6.5	5.0E-03
SH3BGRL2	↓	3.2	1.9E-06	↓	52.0	5.0E-03
SLC16A3	↑	2.1	4.3E-04	↑	1.6	4.7E-02
CXCL10	↑	16.5	1.3E-07	↑	34.9	4.7E-02

a Wilcoxon matched pairs signed rank test.

## Discussion

Oral cancer is a relatively aggressive disease and the five year survival of approximately 50% has not improved over the last few decades regardless of significant improvements in surgery and radiotherapy [24], [25]. Early spread to local lymph nodes and failure of treatments are two major reasons for the poor prognosis [24], [26]. Identification of novel biomarkers for early detection and prediction of response to treatment, as well as for use as treatment targets is of utmost importance to increase survival in this patient group.

Transcriptional profiling has great potential in the search for new biomarkers and treatment targets but has been limited by the low number of fresh frozen samples available to ask specific questions of specific cancer sub-types. With the new technologies enabling analysis of partially degraded RNA from stored FFPE material new possibilities are emerging [13], [14]. Here, we analysed global gene expression in FFPE sample from a subgroup of oral cancer patients with tumours on the tongue. We, like others, observed reasonable sensitivity with on average 52% detected genes and very good reproducibility (r^2^ = 0.97–0.98) using the WG DASL assay [14], [18]–[20]. Perhaps not surprisingly, the performance of the arrays was dependent on the quality of the RNA and 70% of the variation in number of genes detected in a sample is explained by the comparative amplification of a control house-keeping mRNA (Ct_diff_). A loss of signal with time and quality has been reported before [18], [27]. More worrisome was the finding that the expression levels of individual genes were not affected equally; the expression level of around half of the detected genes was influenced by Ct_diff_. Traditional methods for normalizing microarray data assume that most mRNAs are affected similarly in each sample. Our data, however, contradict this assumption for FFPE material and indicate that current methods used to normalize microarray data will not remove the variation caused by a difference in quality of these samples.

The reason for non-random effects in RNA extracted from FFPE material is likely to relate to the observations that RNA is not only degraded in FFPE sample but is also modified. These modifications have been shown to affect different nucleotides to varying extents and will disrupt cDNA and PCR reactions [11], [18], [28]. In addition, oxidation of nucleotides may accrue over time and is also sequence dependent. Mittempergher et al. previously reported a higher concordance between FFPE and FF material for probes with a high GC content. We observed a higher number of Cytosines in genes whose expression is least influenced by sample quality while the number of Guanines are lower, confirming that sequence can provide information about which genes are more reliably detected and quantified.

Variation in RNA stability is another factor likely to be important for individual genes being affected differently by sample quality and has also been suggested to be partially sequence dependent [29]. In our data, the supposedly more stable small RNAs [30], snoRNA and miRNA, had a distinct expression pattern as compared to longer RNAs. Whereas the expression of most of the affected RNAs decreased with sample quality the expression of many of the smaller RNAs increased. One possible explanation for this could be a change in composition of total RNA in the extract from these samples; while the more unstable larger RNAs are lost with poorer quality the fraction of the more stable small RNAs will increase. These results show that identifying the genes most affected by sample storage and the mechanisms involved in RNA degradation and modification in FFPE samples may help improve the design of future arrays and the development of FFPE-specific normalization steps, leading to more sensitive analysis of global gene expression in FFPE samples.

When reducing the number of samples analyzed so that both controls and tumours were of the same quality range we could show that the difference between tongue tumours and control samples produced the largest variation within the data set, as shown in the unsupervised cluster analysis. A total of 934 genes were significantly differentially expressed in tumours and their biological relevance to carcinogenesis was confirmed using pathway analysis. Not many whole genome studies on tongue SCC have been performed previously but Ye et al. presented a 35 gene profile based on RNA from fresh frozen material [31]. In spite of the differences in sample types and platforms used we could confirm the majority of these genes in our data, 15 of the 17 up-regulated genes and ten of the 18 down-regulated genes. To further confirm the validity of our data set, four genes that we identified that have not previously been connected to tongue SCC were confirmed in a set of fresh frozen tissues. Two of these novel genes, SLC2A6 and SLC16A3 are membrane-bound transporters with roles in energy metabolism. SLC2A6, also known as GLUT6, is a member of the solute-linked carrier gene family SLC2 of facilitative glucose transporters. GLUT6 has an ill-defined function but has been found to be dysregulated in chronic lymphocytic leukaemia and up-regulation of glucose transporters has been reported in many cancer types [32], [33]. SLC16A3 (also known as MCT4) is a proton-coupled lactate transporter that is responsible for removing excess lactate from tumour cells resulting from their increased usage of glycolysis and recent data indicate that MCT4 directly regulates the growth of cancer cells [33], [34]. Another gene we identified is SH3BGRL2 which belongs to a newly discovered family of thioredoxin-like proteins [23]. We found a large down-regulation of SH3BGRL2 mRNA levels and even though the other two family members, SH3BGRL and SH3BGRL3, were not significantly down-regulated both showed lower expression in tumours (−1.7 and −3.2 fold respectively) indicating that the whole family may be decreased in tumours. Although very little is currently known about the function of these proteins, SH3BGRL down-regulation has been shown to be important for v-Rel-mediated transformation [35]. Finally, we confirmed an up-regulation of the pro-inflammatory chemokine CXCL10 which is expressed by various cancer cell types and influences tumour progression through the recruitment of specific immune cell types into the tumour microenvironment, and has potential as an immunotherapeutic approach [36], [37].

In conclusion, we show that analyzing FFPE samples using the whole genome DASL array can generate highly informative results but needs to be performed with care. Although the method is highly reproducible, expression levels are significantly influenced by sample quality in a manner that relates to individual RNA probe sequences. This will impair normalization and lead to a residual non-biological variation within the data. In order to minimize the false detection rate and to maximize the level of biologically relevant information obtained, samples should be of the same quality range in groups to be compared. Thus, we recommend that such studies be performed using only samples that are matched for their quality following an initial qPCR reaction and that samples with discordant results are excluded from further analysis. In practice, this means that a larger number of samples than ultimately required need to be screened for quality assessment. Given the vast number of archival samples available, this does not represent a major problem for sample acquisition. In addition, we recommend that results are independently confirmed at an early stage, for example by the use of qPCR on FF material from a more limited number of samples. We show that by taking these simple precautions gene lists obtained from FFPE material will be of high biological relevance and we used it both for confirming a previous result [31] as well as for making novel findings that were confirmed in high quality fresh frozen samples. Limitations of FFPE material can thus be reduced by careful selection of samples with adequate quality. Given the huge numbers of patient samples potentially available and the relative ease of quality assessment, there is an opportunity for archival material to identify novel pathways and biomarkers in common and rare cancers and their subtypes.

## Supporting Information

Table S1 **Significantly differentially expressed genes between cancer and control samples.** Table of differentially expressed genes from analysing the 43 selected samples. Table includes average signal for the two groups, p values and fold changes.
(XLSX)
